# Assessment of Nurses' Practice and Potential Barriers Regarding the Medical Waste Management at Hamad Medical Corporation in Qatar: A Cross‑Sectional Study

**DOI:** 10.7759/cureus.8281

**Published:** 2020-05-25

**Authors:** Fatema Musa, Ayatullah Mohamed, Nagah Selim

**Affiliations:** 1 Epidemiology and Public Health, Primary Health Care Corporation, Doha, QAT; 2 Community Medicine, Hamad Medical Corporation, Doha, QAT; 3 Community Medicine, Primary Health Care Corporation, Doha, QAT

**Keywords:** waste management, nurses, practice, barrier, qatar

## Abstract

Background

The improper management of wastes generated in healthcare facilities can severely affect the health of caregivers, patients, and members of the community. Medical waste management can be achieved with the cooperation of all workers and patients; however, nursing personnel plays a significant role in the whole process^.^ Therefore, nurses need to be well equipped with skills and practices in managing medical waste. This will result in the reduction of risks and hazards to their health. This study is done to assess the practice of nursing professionals regarding waste management across Hamad Medical Corporation (HMC) hospitals in Doha and to identify the potential barriers toward medical waste management

Methods

An analytical cross‑sectional study conducted at four governmental hospitals in Doha city; Hamad General Hospital (HGH), Women's Hospital (WH), Rumiallalh Hospital (RH), and Al-Amal hospital. A stratified proportionate random sampling method was employed to recruit 420 nurses.

Results

The response rate among nurses was 82.3%, with most of them being females and non-Qatari. Overall, the correct practice of color-coding of different waste categories among nurses was 92.8%. Unavoidable exposure was identified by 60.3% of nurses as a barrier to waste management, and nurses working at the intensive care unit reported it at the highest percentage (67.2%)

Conclusions

The majority of nurses showed correct practice and could correctly match the color-coding of different waste categories. Unavoidable exposure and excessive production of waste were the most reported barriers. Excessive production of waste and unavoidable exposure should be further evaluated by quantifying medical waste and addressing appropriate control strategies tackling the identified barriers.

## Introduction

Improper management of wastes at healthcare facilities is lethal and can have health impacts on the community, the personnel working in these facilities, and the environment [1]. The term "healthcare waste" (HCW) includes all the waste generated within healthcare facilities, research centers, and laboratories related to medical procedures. Also, it includes the same types of waste originating from minor and scattered sources, including waste produced in the course of healthcare undertaken in the home (e.g. home dialysis, self-administration of insulin, recuperative care [2].

According to the World Health Organization (WHO), around 85% of hospital wastes are non-hazardous, the remaining 15% is considered hazardous material that may be infectious, chemical, or radioactive [3]. Segregation is one of the most important steps to successfully manage HCW. Given the fact that only about 10-25% of the HCW is hazardous, treatment and disposal costs could be greatly reduced if proper segregation was performed. Also, segregating hazardous from non-hazardous waste greatly reduces the risk of infecting workers handling HCW. The part of the HCW that is hazardous and requires special treatment could be reduced to some 2-5% if the hazardous part was immediately separated from the other waste. The application of a color-coding system aims at ensuring an immediate and non-equivocal identification of the hazards associated with the type of HCW that is handled or treated. In that respect, the color-coding system should remain simple and be applied uniformly throughout the country [4].

The different types of waste are sorted and known as follows: infectious waste: waste contaminated with blood and other bodily fluids from laboratory work or patients with infections; pathological waste: human tissues, organs or fluids, body parts, and contaminated animal carcasses; sharps waste: syringes, needles, disposable scalpels and blades; and non-hazardous or general waste: waste that doesn’t pose any biological, chemical, radioactive, or physical hazard [5].

Unsafe injections were responsible for as many as 33,800 new HIV infections, 1.7 million hepatitis B infections, and 315,000 hepatitis C infections. The patient has risks of 30%, 1.8%, and 0.3%, respectively, of becoming infected with HBV, HCV, and HIV [5]. The International Labor Organization (ILO) estimated up to 2.78 million people die from occupation‑related disease and injuries, and 374 million experience non-fatal injuries [6]. The most common problems connected with medical waste include the absence of proper waste management, lack of awareness about the health hazards from medical wastes, insufficient financial and human resources, and poor control of waste disposal [3].

In the state of Qatar, medical waste management is done by different sectors including the Ministry of Municipality and Environment, Ministry of Public Health, and infection control department at Hamad Medical Corporation (HMC). The three sectors are corporate in establishing legislation, policies, and supervising the process of medical waste management and disposal.

Nurses are at higher risk of exposure to medical waste hazards. They spend maximum time with patients in the ward than any other member of the health team, increasing their exposure to the risk of hazards. They are also responsible for preventing the risk of waste to the other members of the health team and community at a larger perspective [7]. On the other hand, practice and barriers among nurses regarding medical waste management have not been studied. Thus, this study aims to assess the practice of nursing professionals regarding waste management in Hamad Medical Corporation (HMC) hospitals in Doha and to identify the potential barriers of medical waste management.

## Materials and methods

Methods

This is an analytical cross‑sectional study.

Study settings

This study was conducted across four governmental hospitals in Doha city: Hamad General Hospital (HGH), Women's Hospital (WH), Rumiallalh Hospital (RH), and Al-Amal hospital. The four hospitals are working under HMC, providing secondary and tertiary health services to most of the population in Qatar. It is the premier non-profit main healthcare provider in Doha.

Study population

The study included nursing staff working at the aforementioned hospitals. They were recruited and distributed according to their qualification and experience. The fitness of nurses for work was assured through a preplacement medical examination. The estimated number of nurses working within the HMC at the time of the study was 5316 nurses working at administration and health information systems. Those on leave during the data collection were excluded from the study sampling frame.

Sampling technique

A stratified random sampling method with proportionate allocation was utilized, where four hospitals within Doha city were included in the study. A list of all nurses working in these hospitals was obtained by the investigator from the human resources section of HMC. Using this list, the investigator applied the sampling method in proportion to the total number of nurses in each hospital. The estimated sample size was 420 nurses using a 95% confidence level and 5% absolute precision, the effect size of 50%, and 20% added for non-response.

A modified, validated and an anonymous questionnaire was employed for data collection. It comprised demographic data including age, gender, nationality, educational level, place of work, working area, departments, and working experiences, in addition to the questionnaire to assess their practice regarding the segregation of waste at the time of generation, the color-coding of medical waste with a specific color, and the potential barriers. The questionnaire was pre-tested for question variation, meaning, difficulty, respondent interest, and attention.

Data management plan

Statistical Package of Social Science (SPSS), version 16 was used. Descriptive and analytic statistics were used as appropriate. Statistical significance is considered at *p* ≤ 0.05.

## Results

The response rate was 82.3%. Approximately, half of the participating nurses (49.9%) were working at inpatient hospital wards followed by 17.7% at intensive care units, while operating theatres included 4.3% of them, with mostly female and non-Qatari (Table 1)

**Table 1:** Frequency distribution of the background characteristics of nurses (N = 345) HGH, Hamad General Hospital; WH, Women's Hospital; RH, Rumiallalh Hospital

Variable	Frequency	
	No.	%
Age Group		
≤30	77	22.3
31–35	85	24.6
36–40	87	25.2
>40	96	27.8
Gender		
Male	30	8.7
Female	315	91.3
Nationality		
Qatari	7	2.0
Non-Qatari	338	98.0
Educational level		
Secondary	39	11.3
High Nursing institute	109	31.6
University & Post Graduate	197	57.1
Name of hospital		
HGH	169	49.0
WH	78	22.6
RH	76	22.0
Al-Amal Hospital	22	6.4
Duration of work		
1–10 years	148	42.9
11–20 years	149	43.2
21 years and above	48	13.9
Workplace		
Outpatient clinic	29	8.4
Intensive Care Unit	61	17.7
Inpatienthospital ward	172	49.9
Emergency Unit	35	10.1
Operating Theatre	15	4.3
Others	33	9.6

**Figure 1:**
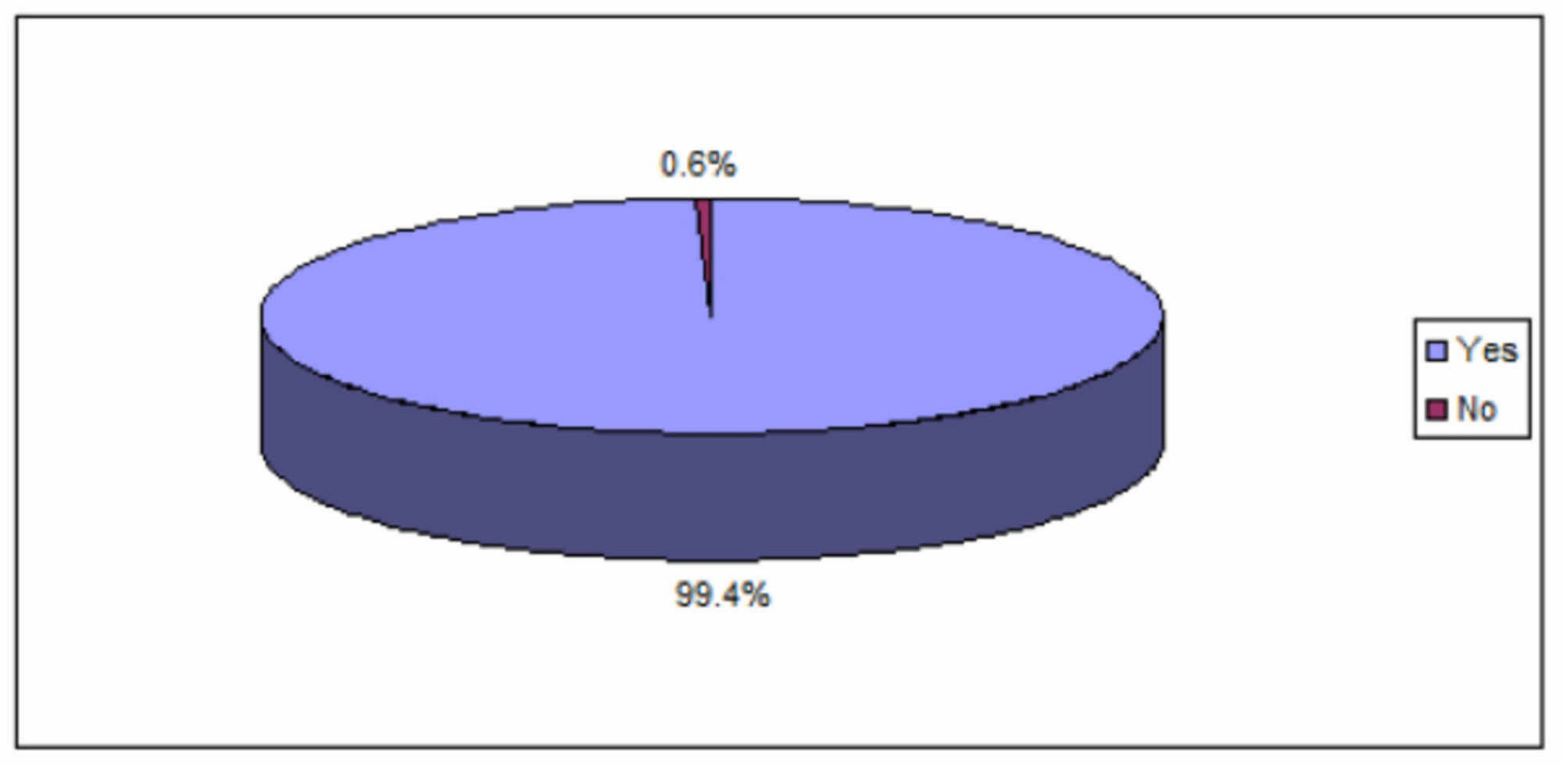
Nurses segregation practice of medical waste at the point of generation into different categories (N = 345)

Regarding the segregation of medical waste at the point of generation into different categories, Figure 1 shows that there was an overwhelming majority (99.4%) reporting that they segregate the waste at the point of generation into different categories, while the rest of the nurses (0.6%) do not involve in the process of segregation.

The frequency distribution of correct practice regarding color-coding of different waste categories is shown in Table 2. 

**Table 2:** Frequency distribution regarding color-coding practice of different waste categories among studied nurses (N = 345)

Incorrect		Correct		Waste Categories
%	No.	%	No.	
1.2	4	98.8	341	Infectious Waste (sharps)
1.2	4	98.8	341	Infectious Waste (non-sharps)
29.9	72	79.1	273	Chemical Waste (sharps)
27.4	74	78.6	271	Chemical Waste (non-sharps)
45.8	158	54.2	187	Radiological Waste
2	7	98.0	338	Domestic Waste
74.9	293	15.1	52	Pathological Waste

For infectious waste (sharps and non-sharps), the majority of participant nurses (98.8%) matched the waste categories with color-coding. Domestic waste had a considerable percentage (98%) of correct practice reported by the nurse, and finally, pathological waste had the lowest percentage (15.1%) of correct practice among all waste categories of matching color-coding.

**Figure 2:**
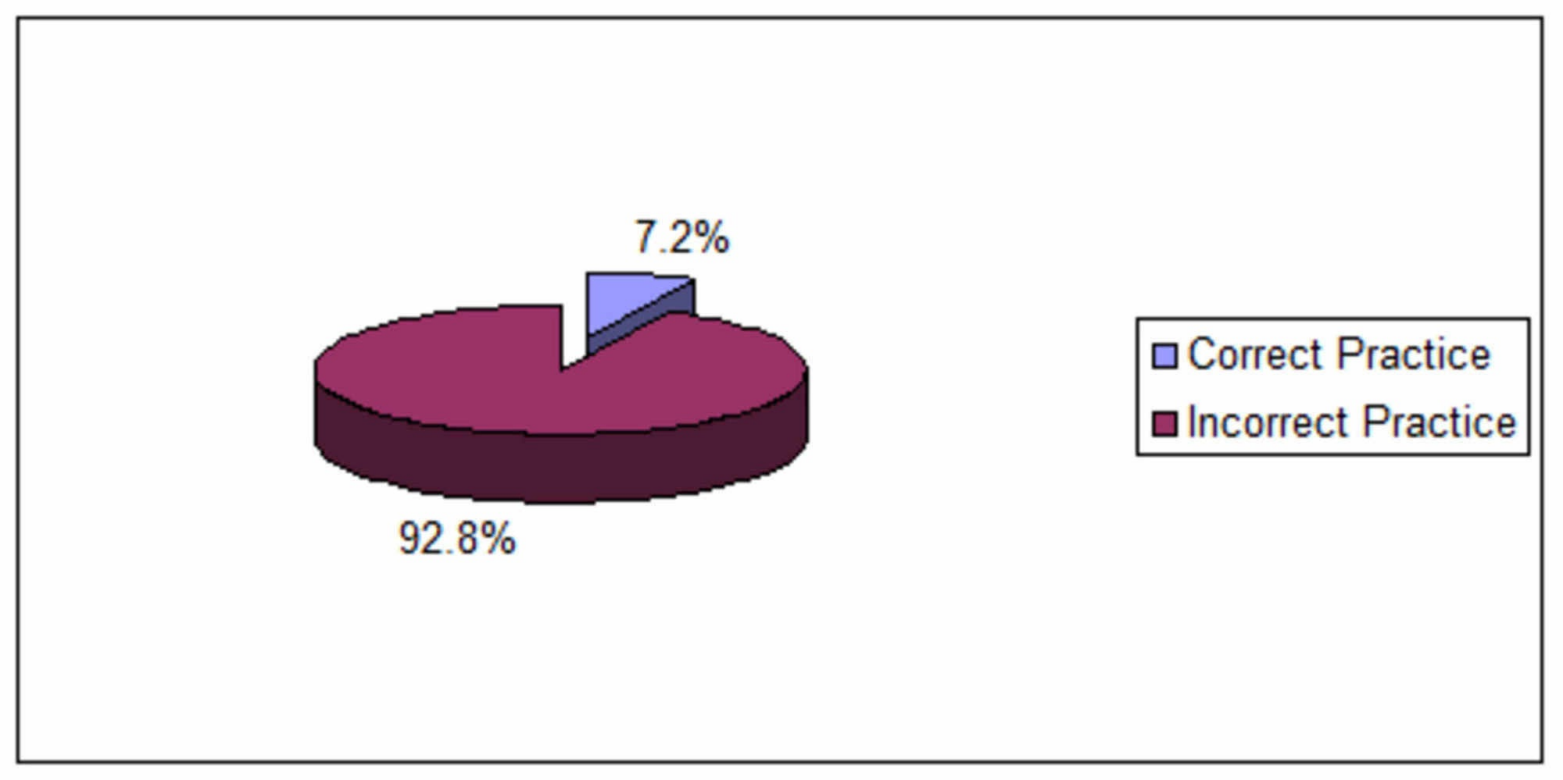
Overall correct practice of color-coding of different waste categories among nurses (N = 345)

Figure 2 shows the frequency distribution of studied nurses regarding the overall correct practice of color-coding of different waste categories. The majority of nurses (92.8%) were practicing incorrect color-coding for the different waste categories, while only 25 participants (7.2%) of nurses were able to match the color code of all waste categories correctly.

**Figure 3:**
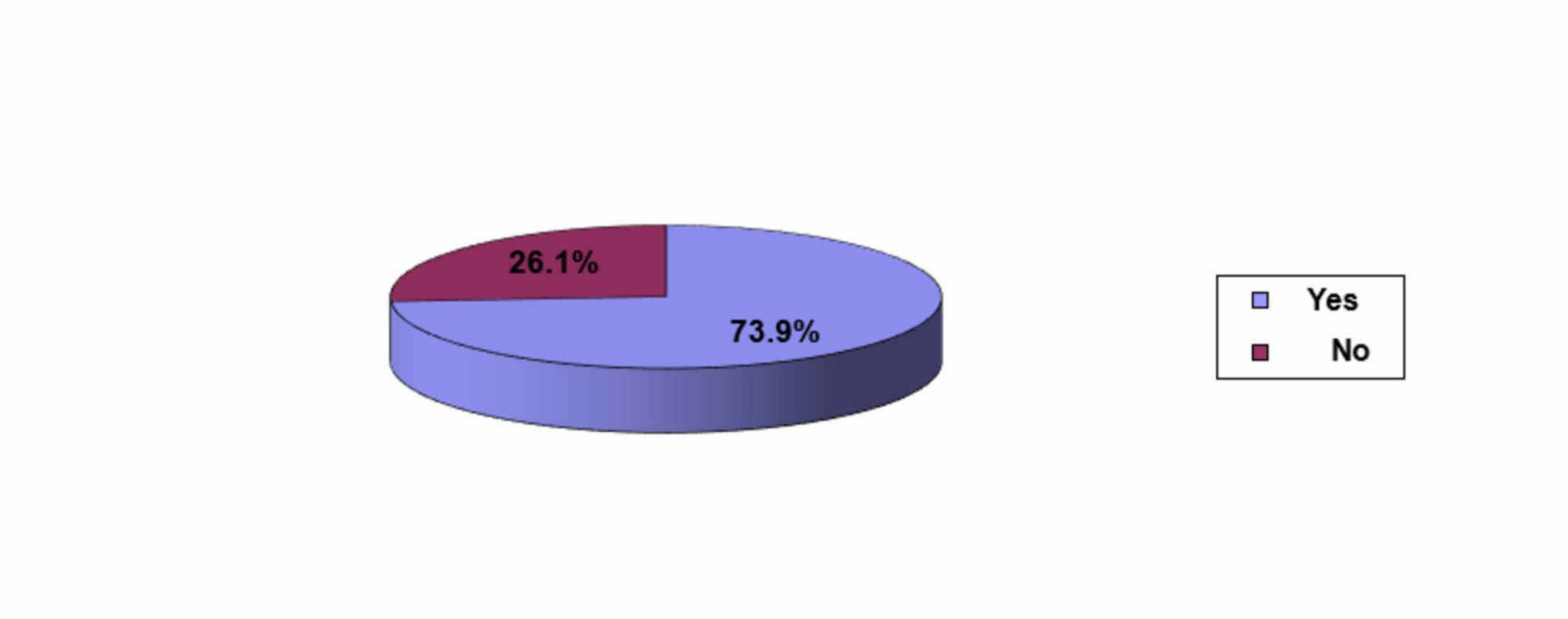
Undergoing training programs on medical waste management among nurses (N = 345)

The frequency distribution of studied nurses regarding if they had undergone training programs on medical waste management is provided in Figure 3. Data on nurses training showed that almost three quarter (73.9%) of participating nurses had undergone training programs.

The frequency distribution of studied nurses regarding barriers of medical waste management is explained in Table 3. 

**Table 3:** Frequency distribution of studied nurses regarding barriers toward medical waste management (N = 345)

Practice	Yes		No	
	Frequency	%	Frequency	%
Unavoidable exposure	208	60.3	137	39.7
Excessive production of waste	137	39.7	208	60.3
Lack of personal protective equipment	95	27.5	250	72.5
Inadequate special containers for disposal	84	24.3	261	75.7
Lack of training	112	32.5	233	67.5
Lack of guidelines and legislations	89	25.8	256	74.2
Time constrains	104	30.1	241	69.9

Data on barriers that may face the nurses in the management of medical waste indicate that a considerable percentage of nurses (60.3%) regard the unavoidable exposure as one of those barriers. On the other hand, the inadequacy of special containers for waste disposal attained the lowest percentage (24.3%) among the studied nurses.

The current study showed non-statistical significance between the overall correct practice of color-coding of different waste categories and the duration of working experience (Table 4).

**Table 4:** Overall correct practice of color-coding of different waste categories according to duration of working experience of nurses (N = 345)

Duration of Working Experience	Overall Correct Practice of Color-Coding				p-Value
	Correct		Incorrect		
	No	%	No	%	
1-10 years	9	36	139	43.4	0.138
11-20 years	15	60	134	41.9	
21 years and above	1	4	47	14.7	

Table 5 shows that about two-third of nurses working at outpatient clinics, intensive care units, and inpatient hospital wards reported the unavoidable exposure as one of the barriers facing the management of medical waste (62.1%, 67.2%, and 64%, respectively) with a *p*-value of 0.045 indicating statistically significant results and suggesting a relationship between unavoidable exposure as a barrier in the medical waste management and working settings of nurses.

**Table 5:** Barriers of medical waste management according to working place of nurses (N = 345)

Barriers	Working place												p-Value
	Outpatient Clinic		Intensive Care Unit		Inpatient Hospital Ward		Emergency		Operating Theatres		Others		
	Yes	No	Yes	No	Yes	No	Yes	No	Yes	No	Yes	No	
	No. (%)	No. (%)	No (%)	No (%)	No (%)	No. (%)	No. (%)	No. (%)	No. (%)	No.(%)	No. (%)	No. (%)	
Unavoidable exposure	18(62.1)	11(37.9)	41(67.2)	20(32.8)	110(64)	62(36)	20(57.1)	15(42.9)	5(33.3)	10(66.7)	14(42.4)	19(57.6)	0.045
Excessive production of waste	5(17.2)	24(82.8)	30(49.2)	31(50.8)	66(38.4)	106(61)	22(62.9)	13(37.1)	4(26.7)	11(73.3)	10(30.3)	23(69.7)	0.002
Lack of personal protective equipment.	11(37.9)	18(62.1)	17(27.9)	44(72.1)	53(30.8)	119(69.2)	6(17.1)	28(82.9)	1(6.7)	14(93.3)	7(21.2)	26(78.8)	0.139
Inadequate special containers for disposal	10(34.5)	19(65.5)	18(29.5)	43(70.5)	39(22.7)	133(77.3)	9(25.7)	26(74.3)	2(13.3)	13(86.7)	6(18.2)	27(81.8)	0.485
Lack of training	12(41.4)	17(58.6)	21(34.4)	40(65.6)	59(34.3)	113(65.7)	8(22.9)	27(77.1)	5(33.3)	10(66.7)	7(21.2)	26(78.8)	0.440
Lack of guidelines and legislations	10(34.5)	19(65.5)	15(24.6)	46(75.4)	46(26.7)	126(73.3)	7(20)	28(80)	4(26.7)	11(73.3)	7(21.2)	26(78.8)	0.813
Time constraints	4(13.8)	25(86.2)	20(32.8)	41(67.2)	58(33.7)	114(66.3)	8(22.9)	27(77.1)	5(33.3)	10(66.7)	9(27.3)	24(72.7)	0.305

About two-third of nurses (62.9%) working at the emergency unit reported excessive production of waste as one of the barriers facing the medical waste management being the highest percentage achieved among all working settings and the lowest percentage was achieved by nurses working at an outpatient clinic (17.2%). The *p*-value was estimated to be 0.002, pointing out a relation between excessive production of waste as a barrier facing the management of medical waste and working settings of nurses.

## Discussion

The percentage of female nurses was found to be high (91.3%) compared to male nurses (8.7%); women are commonly the predominant gender in many healthcare settings. In Oman, males represent the proximity of 4% of the registered nurse population, which is consistent with the demographic nurse population in the current study [8]. More than half of the study participants (57.1%) attained a university and postgraduate degree, and 11.3% of nurses had secondary school-level education which represents the policy.

Regarding the segregation of medical waste at the point of generation into different categories, an overwhelming majority of nurses (99.4%) had reported that they segregate waste at the point of generation into different categories. Incomparable to study done in Ethiopia, 2018 among health care workers in tertiary hospitals, 275 (92.9%) of the health-care workers segregated the waste at the source of generation [9].

Data on nurses training and education showed that most of the nurses almost (74%) attended training regarding waste management, and this high percentage is accomplished through different ways including memos, announcements, and the HMC website which announces all training programs and workshops and sends emails to each employee addressing any upcoming courses and workshops. Similarly, in a study done in Bangladesh, 2014, 61.6% had trained in hospital waste management, while 38.4% did not have training [10]. This is in contrary to the study in Iraq in 2016 among nurses where training and workshop attending was only 15% [11].

The barriers to medical waste management among nurses were pointed out. Data on barriers that may face the nursing staff in the management of medical waste indicate that a considerable percentage of nurses (60.3%) regard the unavoidable exposure as one of the barriers.

Lack of guidelines and legislation regarding medical waste management was obtained by 25.8% of participating nurses. On the other hand, inadequate special containers for waste disposal attained the lowest percentage (24.3%). The low frequency of nurses reporting lack of guidelines and legislation and containers of medical waste disposal may be related to the fact that HMC had a clear guideline and legislation concerning medical waste management and had provided all health-care facilities with appropriate containers, bins, and bags designed for disposal and management of medical waste.

The study carried out in Bangladesh in 2014 also reported possible barriers in managing medical waste among nurses including lack of guideline/policy (50.9%), lack of vaccination program for health-care providers (46.4%), insufficient personal protective measures (PPE) in the hospital (69.5%), lack of instrument for final disposal (56.4%), and insufficient recycle bin/container (30.5%). Their studies then recommended that the need for formulating rules and guidelines for medical waste management should be ensured [12].

Also, in a study done in Botswana, the possible barriers reported in managing medical waste among nurses included lack of knowledge (63%), lack of training as the best practice of waste management (52.7%), nurses don’t see waste disposal as their concern (52.2%), and lack of color-coding material such as plastic bags (29%) [13].

The relation between the overall correct practice of the color-coding of the different waste categories according to working experience and place of work of nurses was investigated. It was found that there was no statistically significant relationship between the overall correct practice of color-coding of different waste categories and the working experience of nurses. This may be explained by the fact that the nursing staff gains their practice through daily working and managing medical waste regardless of their education, years of working, and the place of work.

Another reason is that all healthcare staff was uniformly provided with the same training program addressing medical waste management and infection control measures as routine activity by the corporation, either pre-service on their recruitment or as in-service training regardless of their demographics characteristics.

This result is in line with the study done in Kenya in 2016: there was no significant difference among health-care workers and no statistically significant relationship between the overall correct practice of color-coding of different waste categories and the profession, level of education, and job experience [14]. Contrarily, a study done in Tehran showed that the practice of health-care regarding medical waste management was statistically significant in relation to the level of education [15].

The relation between barriers of medical waste management according to working settings was also examined. It was found that about two-thirds of nurses working at intensive care unit, inpatient hospital wards, and outpatient clinics reported the unavoidable exposure as one of the barriers facing the management of medical waste (67.2%, 64%, and 62.1%, respectively) with statistically significant results, suggesting a relationship between unavoidable exposure as a barrier facing the medical waste management and working setting of nurses.

Nurses working in the intensive care unit reported the highest percentage for unavoidable exposure (67.2%). This may due to that waste resulted from intensive medical care is classified as infectious in nature and the procedures for handling, treatment, and disposal of this waste require to comply with the most stringent standards, which increase the probability of unavoidable exposure to these types of waste.

Regarding the excessive production of waste as a barrier to medical waste management, it was most frequently reported by nurses in the emergency unit (62.9%) and least frequently reported by nurses in the outpatient clinic (17.2%). The relation between excessive production of waste as a barrier to the management of medical waste and the working setting of nurses is statistically significant.

Excessive production of waste by emergency can by elaborated by that emergency unit uses a large amount of disposable material in the care process which rapidly increases the amount of waste in addition to the large number of patients visiting this unit daily reflecting the capacity of services provided by this setting.

## Conclusions

The majority of participating nurses had correctly matched the color-coding of different waste categories. Unavoidable exposure and excessive production of waste were the most identified barriers. The excessive production of waste and unavoidable exposure should be further evaluated by quantifying medical waste at each hospital setting and identifying appropriate control strategies tackling these barriers. It is also important to provide educational materials addressing the color-coding of different waste categories in the form of leaflets and posters in all hospital settings.
